# Cross-neutralizing antibody against emerging Omicron subvariants of SARS-CoV-2 in infection-naïve individuals with homologous BNT162b2 or BNT162b2(WT + BA.4/5) bivalent booster vaccination

**DOI:** 10.1186/s12985-024-02335-9

**Published:** 2024-03-21

**Authors:** Samuel M.S. Cheng, Chris K.P. Mok, John K.C. Li, Ken K.P. Chan, Kristine S. Luk, Ben H.W. Lee, Haogao Gu, Karl C.K. Chan, Leo C.H. Tsang, Karen Y.S. Yiu, Ken K.C. Ling, Yun Sang Tang, Leo L.H. Luk, Jennifer K.M. Yu, Andrew Pekosz, Richard J. Webby, Benjamin J. Cowling, David S.C. Hui, Malik Peiris

**Affiliations:** 1 School of Public Health, LKS Faculty of Medicine, The University of Hong Kong, Hong Kong SAR, China; 2 The Jockey Club School of Public Health and Primary Care, The Chinese University of Hong Kong, Hong Kong SAR, China; 3 Li Ka Shing Institute of Health Sciences, Faculty of Medicine, The Chinese University of Hong Kong, Hong Kong SAR, China; 4 Department of Medicine & Therapeutics, Faculty of Medicine, The Chinese University of Hong Kong, Hong Kong SAR, China; 5 Princess Margaret Hospital, Hospital Authority, Hong Kong SAR, China; 6 W. Harry Feinstone Department of Molecular Microbiology and Immunology, The Johns Hopkins Bloomberg School of Public Health, Baltimore, MD USA; 7 Department of Infectious Diseases, St. Jude Children’s Research Hospital, Memphis, TN USA; 8 SH Ho Research Centre for Emerging Infectious Diseases, Faculty of Medicine, The Chinese University of Hong Kong, Hong Kong SAR, China; 9 Centre for Immunology and Infection, Hong Kong Science Park, Hong Kong SAR, China

**Keywords:** SARS-CoV-2, Omicron subvariant, Immune evasion, Neutralization, Antigenic cartography, Bivalent RNA vaccine, Infection-naïve

## Abstract

Since the emergence of SARS-CoV-2, different variants and subvariants successively emerged to dominate global virus circulation as a result of immune evasion, replication fitness or both. COVID-19 vaccines continue to be updated in response to the emergence of antigenically divergent viruses, the first being the bivalent RNA vaccines that encodes for both the Wuhan-like and Omicron BA.5 subvariant spike proteins. Repeated infections and vaccine breakthrough infections have led to complex immune landscapes in populations making it increasingly difficult to assess the intrinsic neutralizing antibody responses elicited by the vaccines. Hong Kong’s intensive COVID-19 containment policy through 2020–2021 permitted us to identify sera from a small number of infection-naïve individuals who received 3 doses of the RNA BNT162b2 vaccine encoding the Wuhan-like spike (WT) and were boosted with a fourth dose of the WT vaccine or the bivalent WT and BA.4/5 spike (WT + BA.4/5). While neutralizing antibody to wild-type virus was comparable in both vaccine groups, BNT162b2 (WT + BA.4/BA.5) bivalent vaccine elicited significantly higher plaque neutralizing antibodies to Omicron subvariants BA.5, XBB.1.5, XBB.1.16, XBB.1.9.1, XBB.2.3.2, EG.5.1, HK.3, BA.2.86 and JN.1, compared to BNT162b2 monovalent vaccine. The single amino acid substitution that differentiates the spike of JN.1 from BA.2.86 resulted in a profound antigenic change.

## Introduction

SARS coronavirus 2 (SARS-CoV-2) Omicron subvariants (Phylogenetic Assignment of Named Global Outbreak lineage B.1.1.529) with extensive spike protein mutations emerged at the end of 2021 and showed marked escape from neutralizing antibody elicited by past infection or vaccination, although three doses of Comirnaty BNT162b2 Pfizer, Mainz, Germany/New York, United States) vaccine restored neutralizing antibody to presumed protective levels [1]. Further variants have continued to emerge with progressively increasing immune evasion and transmissibility. To address this progressive antibody evasion, a bivalent vaccine expressing the original SARS-CoV-2 (WT) as well as Omicron subvariant BA.4/5 spike was developed and used with the aim of restoring serum neutralizing activity. Booster doses with bivalent vaccines elicited higher neutralizing antibody titres to newer variants compared to monovalent boosters based on the original Wuhan-like (WT) virus spike and those with breakthrough infection and bivalent vaccines had even higher levels of neutralizing activity against Omicron subvariants [2, 3]. As newer Omicron subvariants such as XBB.1.5, XBB.1.16, XBB.19.1, XBB.2.3.2, EG.5.1, HK.3, BA.2.86 and JN.1 continue to emerge, it remains important to monitor evasion from vaccine-elicited neutralizing antibody. However, given that population immunity is increasingly confounded by natural infection with various SARS-CoV-2 variants, it is becoming increasingly difficult to assess the intrinsic immunogenicity elicited by the vaccines alone [1–3].

An RNA vaccine BNT162b2 and an inactivated whole-virus vaccine (CoronaVac, Sinovac Biotech Ltd, Beijing, China) were the two vaccines available in Hong Kong from early 2021. BNT162b2(WT + BA.4/5) bivalent vaccine was available in Hong Kong in late 2022. Because Hong Kong effectively contained SARS-CoV-2 through 2020–2021 using social and public health measures, with population sero-prevalence levels of < 1% by end 2021, we still had a few infection-naïve individuals who received 4th vaccine doses with monovalent BNT162b2(WT) (*n* = 18) or three doses of monovalent vaccine boosted by bivalent BNT162b2(WT + BA.4/5) immunization (*n* = 8). We used pre and post 4th vaccine dose sera in individuals serologically confirmed to be infection-naïve, to compare the intrinsic immunogenicity of these vaccines in eliciting neutralizing antibodies to Wuhan-like (WT) SARS-CoV-2 and Omicron subvariants BA.5, XBB.1.5, XBB.1.16, XBB.1.9.1, XBB.2.3.2, EG.5.1, HK.3,BA.2.86 and JN.1, unconfounded by prior unsuspected infection.

## Methods

### Serum sample collection

The study was approved by the Institutional Review Board of The Hong Kong University and the Hong Kong Island West Cluster of Hospitals (UW 20–169). All participants had provided signed informed consent. Paired sera were collected before the 4th dose and 1 month after 4th dose of vaccine from infection-naïve individuals who received 4 doses of homologous BNT162b2(BBBB) (*n* = 18) and those receiving 3 doses BNT162b2 (WT) with 4th dose as BNT162b2(WT + BA.4/BA.5) bivalent vaccine (BBB + BIV) (*n* = 8). The vaccinees had no previous history of known SARS-CoV-2 infections and were serologically confirmed to have no prior infection by testing antibody negative to SARS-CoV-2 N protein in an anti-N-CTD IgG ELISA assay which can detect past infection and differentiate natural COVID-19 infection from vaccination [4].

### Virus isolation

SARS-CoV-2 wild type (WT) strain (hCoV-19/Hong Kong/VM20001061/2020), Omicron subvariants, XBB.1.16(hCoV-19/Hong Kong/HKUSPH_VOC1933P3/2023), XBB.1.9.1(hCoV-19/Hong Kong/HKUSPH_VOC1636P3/2023),XBB.2.3.2(hCoV-19/Hong Kong/HKUSPH_VOC1934P3/2023), EG.5.1(hCoV-19/Hong Kong/HKUSPH_VOC2249P3/2023), HK.3 (hCoV-19/HongKong/HKUSPH_DRV0280P3/2023), BA.2.86(hCoV-19/Hong Kong/HKUSPH_LRS0548P3/2023) and JN.1 (hCOV-19/HongKong/HKUSPH_VOC2401P2/2023) were isolated at The University of Hong Kong. Omicron subvariant BA.5 (SARS-CoV-2/human/USA/COR-22-063113/2022) and XBB.1.5(SCV2/USA/MD—HP40900/2022;) were kindly provided by Dr Richard Webby, St Jude Children’s Research Hospital, Memphis, US. Virus stocks were passaged in Vero-E6 (African Green Monkey kidney epithelial) cells expressing the transmembrane serine protease TMPRSS2, aliquoted, stored frozen at -80 °C, virus titres obtained by plaque titrations and used in the plaque reduction neutralization tests. Sequences of the viruses used are available in GISAID as EPI_ISL_408975, EPI_ISL_18604375, EPI_ISL_18604376, EPI_ISL_18604377, EPI_ISL_18604378, EPI_ISL_18604484, EPI_ISL_18604485, and EPI_ISL_18888405.

### Plaque reduction neutralization test (PRNT)

Live-virus plaque reduction neutralization tests (PRNT) were performed in duplicate using 24-well tissue culture plates (TPP Techno Plastic Products AG, Trasadingen, Switzerland) in a biosafety level 3 facility using Vero E6 TMPRSS2 cells [5] as previously described [6]. Cells were cultured in Dulbecco’s Modified Eagle Medium (DMEM) medium supplemented with 10% fetal bovine serum (FBS) and 100 U/mL of penicillin-streptomycin. All sera were heat-inactivated at 56 °C for 30 min prior to testing. Serial twofold dilutions from 1:10 to 1:320 of each serum sample were incubated with 30–40 plaque-forming units of virus for 1 h at 37 °C, the virus–serum mix was added onto pre-formed cell monolayers and incubated for 1 h at 37 °C in a 5% CO2 incubator. The virus-antibody inoculum was then discarded, and the cell monolayer was overlaid with 1% agarose in cell culture medium. After 3 days incubation, the plates were fixed with 10% formalin in PBS overnight and stained with 1% crystal violet in ethanol. Antibody titres were defined as the highest serum dilution that resulted in ≥ 50% reduction in the number of virus plaques (PRNT_50_). The average plaque numbers observed in the duplicate dilution-series was used for this computation. Virus back titrations, positive and negative control sera were included in every experiment. Samples with PRNT_50_ titre ≥ 1:320 were further titrated to endpoint.

### Antigenic cartography

Antigenic map construction was performed as previously described [7]. In short, this technique quantifies and visualizes neutralization data as a two-dimensional antigenic map. The antigenic map displays the distance between an antiserum point (S) and an antigen point (A), corresponding to the difference in log2 values between the highest observed titer for antiserum S against any antigen and the titer for antiserum S against antigen A. These distances in the map represent antigenic distance according to the neutralization assay results, with the log2 titer being inversely related to the distance between antigens and antisera. Modified Multidimensional Scaling (MDS) methods help to arrange antigen and antiserum points in the map to best fit the target distances provided by the neutralization data. The resulting map has better resolution due to multiple measurement techniques used to determine the positions of antigens and antisera. The Racmacs package (version 1.2.9.) in R was used to compute antigenic maps under default settings, which included 500 optimizations and a minimum column basis parameter set to “none.”

## Results

The individuals vaccinated with BBBB or BBB + BIV had mean age (SD) of 65 (+/-7.6) and 60 (+/-11.0) yrs respectively; 66.7% and 37.5% being of male sex, respectively. There was no significant difference in pre 4th dose PRNT_50_ GMTs to each virus variant tested (Table 1).

**Fig. 1:**
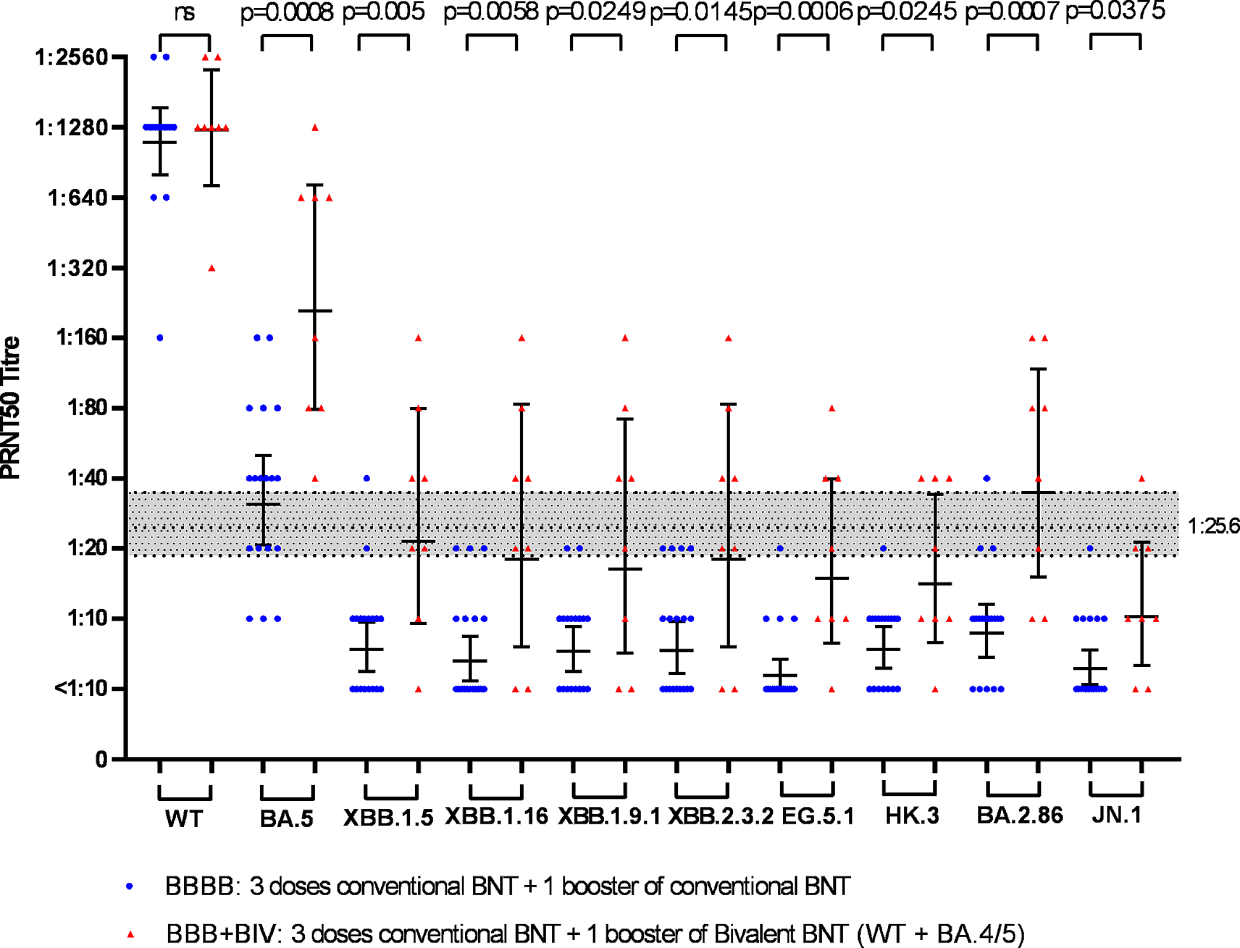
PRNT50 antibody titres to wild-type (WT) SARS-CoV-2 and Omicron subvariants pango lineage BA.5, XBB.1.5, XBB.1.16, XBB.1.9.1, XBB.2.3.2, EG.5.1, HK.3, BA.2.86 and JN.1. Infection-naïve individuals with three doses BNT162b2 boosted by a monovalent WT BNT162b2 (BBBB) vaccine (*N* = 18) are labelled by blue dots, and those boosted with a BNT162b2 bivalent vaccine (BBB + BIV) (*N* = 8) are labelled by red triangles. The Geometric mean titre (GMT) and 95% confidence intervals of GMT are denoted. Mann-Whitney U test was used to test significant differences between the GMTs of the groups. The horizontal dotted line at a titre 1:25.6 represents the 50% protective threshold against symptomatic infection (see text) as defined by Khoury et al. [8–10] and the shaded area represents the 95% confidence interval of this protective threshold

After the 4th vaccine dose, all individuals in the BBBB and BBB + BIV groups had detectable (≥ 1:10) PRNT_50_ antibody to both WT and BA.5 SARS-CoV-2 viruses. The GMT of the two groups to WT virus did not differ significantly but those boosted with the bivalent vaccine (BBB + BIV) had significantly higher GMT to Omicron BA.5 as expected and also to each of the other more recent Omicron subvariants tested (Table 1). In those boosted with the bivalent vaccine (BBB + BIV), 75-100% had detectable (≥ 1:10) PRNT_50_ antibodies to more recent post-BA.5 omicron subvariants compared to only 22-72% in BBBB group (Table 1). A 50% protective threshold neutralizing antibody titre to WT virus has been estimated by Khoury and colleagues [8], and later extended to other variants including Omicron [9, 10]. In our PRNT_50_ assay, this protective titre is estimated to be 1:25.6 (95% CI 18.3–36.0) [6]. Only 0–6% of the BBBB group had “protective” PRNT_50_ titres to XBB.1.5, XBB.1.16, XBB.1.9.1, XBB.2.3.2, EG.5.1, HK.3, BA.2.86, and JN.1 respectively. In comparison, 50% (XBB.1.5), 50%(XBB.1.16), 50%(XBB.1.9.1), 50%(XBB.2.3.2), 38%(EG.5.1), 38%(HK.3), 63%(BA.2.86) and 13%(JN.1) in BBB + BIV group had “protective” PRNT_50_ antibody titres to these viruses (Table 1; Fig. 1). The progressive immune evasion of the post BA.5 subvariants is seen in both BBBB and BBB + BIV boosted groups. In the BBBB group, EG.5 and HK.3 had the greatest fold-reduction in GMT, 188-fold and 144-fold, respectively. Similarly, the BBB + BIV group had 70-fold and 76-fold reductions of neutralizing titres to EG.5 and HK.3, respectively, to EG.5.1 and HK.3 viruses, relative to WT virus. BA.2.86 appeared to have relatively less evasion of neutralization than EG.5 with fold-reductions compared with WT of 119-fold in BBBB and 29-fold in BBB + BIV vaccinees. JN.1 being the descendent lineage of BA.2.86 with one additional amino acid substitution (L455S) had markedly higher evasion of neutralization than BA.2.86, with fold-reductions in GMT compared with WT of 174-fold in BBBB and 108-fold in BBB + BIV vaccinees. GMT to JN.1 had 3.7-fold reduction in neutralization titres compared to BA.2.86 in BBB + BIV vaccinees (Fig. 1; Table 1). The comparative PRNT_50_.

**Table 1:** PRNT50 geometric mean tires to SARS-CoV-2 subvariants in those receiving four doses of BNT162b2 (BBBB)(*n* = 18) and three doses of BNT162b2 followed by BNT162b2 (WT + BA.4/5) bivalent vaccine (BBB + BIV) (*n* = 8)

	WT	BA.5	XBB.1.5	XBB.1.16	XBB.1.9.1	XBB.2.3	EG.5.1	HK.3	BA.2.86	JN.1	
Exposure group	GMT (95% CI) prior to 4th dose										
BBBB	201.6 (122.1-332.9)	9.259 (6.949–12.34)	5.4 (4.831–6.037)	5.4 (4.831–6.037)	5.196 (4.791–5.636)	5 (5–5)	5 (5–5)	6.3 (5.33–7.446)	6.547 (5.507–7.783)	5 (5–5)	
BBB + BIV	174.5 (64.14–474.6)	7.711 (4.17–14.26)	5.453 (4.442–6.692)	5 (5–5)	5 (5–5)	5 (5–5)	5 (5–5)	5.946 (4.547–7.775)	10.91 (5.296–22.45)	5 (5–5)	
P value*	0.6754	0.3717	> 0.9999	0.5569	> 0.9999	> 0.9999	> 0.9999	> 0.9999	0.0697	> 0.9999	
	**After 4th dose**										
	**GMT (95% CI) after booster**										
BBBB	1140 (848.5–1533)	35.64 (23.17–54.8)	8.249 (6.204–10.97)	7.349 (5.609–9.628)	7.937 (6.266–10.05)	8.249 (6.204–10.97)	6.062 (4.973–7.389)	7.937 (6.467–9.741)	9.622 (7.297–12.69)	6.547 (5.31–8.072)	
BBB + BIV	1280 (748.5–2189)	246.8 (84.62–719.5)	28.28 (11.17–71.63)	25.94 (9.312–72.24)	23.78 (8.226–68.77)	25.94 (9.312–72.24)	18.34 (8.358–40.25)	16.82 (8.562–33.03)	43.62 (16.84–113)	11.89 (6.528–21.66)	
P value*	0.609	0.0008	0.005	0.0058	0.0249	0.0145	0.0006	0.0245	0.0007	0.0375	
	**Number (%) with PRNT**_**50**_ **titre ≥ 1:10**										
BBBB	18(100%)	18(100%)	10(56%)	7(39%)	10(56%)	9(50%)	4(22%)	11(61%)	13(72%)	6(33%)	
BBB + BIV	8(100%)	8(100%)	7(88%)	6(75%)	6(75%)	6(75%)	7(88%)	7(88%)	8(100%)	6(75%)	
	**Number (%) with PRNT**_**50**_ **titre ≥ 1:25.6#**										
BBBB	18(100%)	11(61%)	1(6%)	0(0%)	0(0%)	0(0%)	0(0%)	0(0%)	1(6%)	0(0%)	
BBB + BIV	8(100%)	8(100%)	4(50%)	4(50%)	4(50%)	4(50%)	3(38%)	3(38%)	5(63%)	1(13%)	

**Fig. 2:**
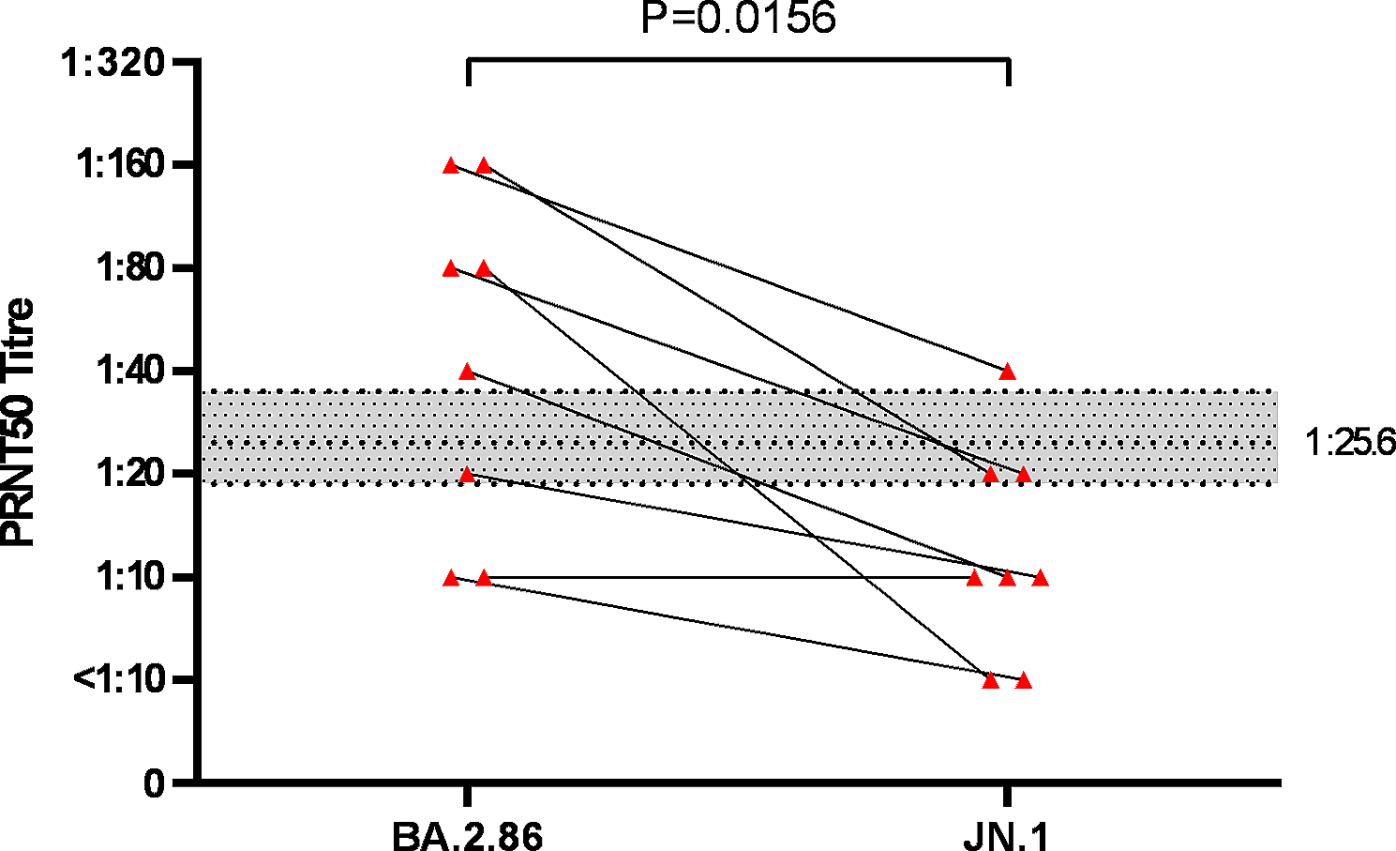
PRNT50 antibody titres to SARS-CoV-2 Omicron subvariants BA.2.86 and JN.1 in BNT162b2 in individuals boosted by a BNT162b2 (WT + BA.4/5) bivalent vaccine (BBB + BIV) (*N* = 8). The PRNT50 titre to BA.2.86 and JN.1 in each individual is linked by a solid line. GMTs between the two groups were tested using Mann-Whitney U test. The horizontal dotted line at a titre 1:25.6 represents the 50% protective threshold against symptomatic infection as defined by Khoury et al. [8] (see text) and the shaded area represents the 95% confidence interval of this protective threshold

*Mann-Whitney test was used to compare GMTs between two vaccine groups. *P* < 0.05 is regarded as significantly different. PRNT_50_ titre 1:10 is the lowest detection limit of the neutralization test. #PRNT^50^ titre 1:25.6 is the 50% protective threshold, computed as defined by Khoury et al. [8]

titres to BA.2.86 and JN.1 in individual sera of those boosted with BBB + BIV vaccine is shown in Fig. 2.

**Fig. 3:**
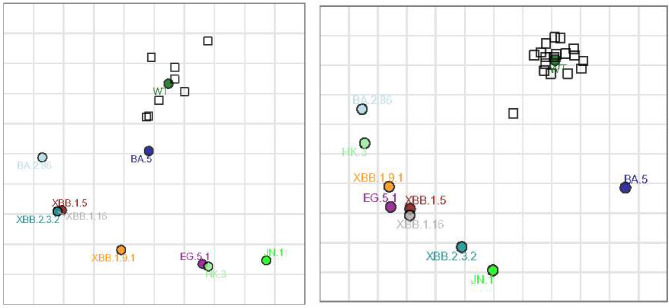
**a** Antigenic cartography using live SARS-CoV-2 viruses in BBB + BIV group (*n* = 8). MDS was used to create an antigenic map from the PRNT50 titers generated against WT, BA.5, XBB.1.5, XBB.1.16, XBB.1.9.1, XBB.2.3.2, EG.5.1, HK.3, BA.2.86, and JN.1 viruses on VeroE6-TMPRSS2 cells. In the map, coloured circles represent viruses, while squares denote antisera. The arrangement of viruses and antisera is such that their distances are inversely proportional to the antibody titers, minimizing error. The background grid corresponds to a twofold dilution of antisera in the titration process. Fold dilution differences between WT and omicron subvariants BA.5, XBB.1.5, XBB.1.16, XBB.1.9.1, XBB.2.3.2, EG.5.1, HK.3, BA.2.86, and JN.1 were 2.3, 5.5, 5.6, 5.7, 5.6, 6.1, 6.2, 4.8, and 6.7 respectively. **b** Antigenic cartography using live SARS-CoV-2 viruses in BBBB group(*n* = 18). MDS was used to create an antigenic map from the PRNT50 titers generated against WT, BA.5, XBB.1.5, XBB.1.16, XBB.1.9.1, XBB.2.3.2, EG.5.1, HK.3, BA.2.86, and JN.1 viruses on VeroE6-TMPRSS2 cells. In the map, coloured circles represent viruses, while squares denote antisera. The arrangement of viruses and antisera is such that their distances are inversely proportional to the antibody titers, minimizing error. The background grid corresponds to a twofold dilution of antisera in the titration process. Fold dilution differences between WT and omicron subvariants BA.5, XBB.1.5, XBB.1.16, XBB.1.9.1, XBB.2.3.2, EG.5.1, HK.3, BA.2.86, and JN.1 are 5.0, 7.1, 7.3, 7.2, 7.1, 7.5, 7.1, 6.9 and 7.4 respectively

Antigenic cartography in these infection-naïve individuals in BBB + BIV group shows WT and BA.5 relatively closer together as expected, while JN.1, EG.5.1 and HK.3 were the farthest away from WT and BA.5 (Fig. 3a). BA.2.86 and JN.1 were antigenically very distant to each other (Fig. 3a, b).

## Discussion

Our study focused on demonstrating the effect of vaccine boosters in infection- naïve individuals, unconfounded by prior infection or vaccine breakthrough infection and thus demonstrates the intrinsic virus neutralizing antibody responses elicited by the vaccine alone. Other studies demonstrated that the BNT162b2(WT + BA.4/5) bivalent vaccine including recent omicron subvariant BA.5 immunogen was superior to monovalent vaccine against omicron subvariants BA.1, BA.2.75.2, BQ.1.1 and XBB.1.5, but these did not exclude individuals with past infection [11, 12]. An approach to understand immune evasion in immune naïve individuals has been to use mono-specific hamster immune sera [7], but hamsters may not fully recapitulate human immune responses. There are fewer reports on immune evasion of JN.1 [13, 14], none of them including infection-naïve individuals. Participants in these studies had past SARS-CoV-2 infection or only excluded subjects with natural infection within 3 months before assessment. We have demonstrated that BBB + BIV booster elicited significantly higher neutralizing antibody to Omicron BA.5 compared to BBBB as expected, but the BBB + BIV also elicited significantly higher PRNT_50_ titres to all the more recent Omicron subvariants tested, including JN.1.

Spike proteins of BA.5, XBB.1.5, XBB.1.16, XBB.1.9.1, XBB.2.3.2, EG.5.1 and HK.3 have over 30 amino acids (a.a.) substitutions relative to WT, while BA.2.86 has over 60 a.a. substitutions relative to WT. Compared to BA.5, the RBD region of XBB.1.5, XBB.1.16, XBB.1.9.1, XBB.2.3.2, EG.5.1, HK.3 and BA.2.86 have additional V445P, G446S, N460K a.a. substitutions which may explain the greater evasion from neutralizing antibody. R346T, L368I and F490S were additional mutations in XBB.1 descendent lineage (XBB.1.5, XBB.1.16, XBB.1.9.1, XBB.2.3.2, EG.5.1, HK.3), but these were not observed in BA.5 or BA.2.86, which may explain the relatively higher neutralizing titres observed with BA.2.86. In our BBB + BIV vaccinated group, the lowest GMT were found with EG.5.1, HK.3 and JN.1 viruses, possibly a result of the F456L mutation. This was also noted in another study which compared the immune evasion of EG.5.1 and XBB.2.3.2 [15]. GMTs to EG.5.1 and HK.3 differed significantly in the BBBB vaccine group which may be due to the L455F mutation in HK.3, a mutation shown by computational analysis to change the protein structure [16]. The greater immune evasion in JN.1 compared to BA.2.86 could be explained by a key mutation L455S in spike protein. This was also demonstrated in other studies by pseudovirus neutralization [13]. However, the difference in neutralizing titres to BA.2.86 and JN.1 was not seen in those recently infected with XBB.1.5 [14].

Our study had a number of limitations including a small sample size, but this was the result of excluding those with prior natural immunity, to allow us to exclusively investigate the intrinsic immunogenicity of the vaccines, unconfounded by prior infection immunity. We used a correlate of protection as described previously [8–10], but we recognize that neutralizing antibody may not be a sole correlate of protection for all vaccines, including inactivated vaccines. Populations in many parts of the world now have complex “hybrid” immune landscapes elicited by vaccination as well as natural infection, making it difficult to extrapolate our data to populations in general but our data are relevant to understanding the intrinsic antibody responses of vaccines. A new monovalent XBB.1.5 vaccine is now being rolled out and it would be important to conduct studies to understand neutralizing antibody responses to this vaccine.

In summary, we assessed the neutralizing antibody to a panel of SARS-CoV-2 subvariants, BA.5, XBB.1.5, XBB.1.16, XBB.1.9.1, XBB.2.3.2, EG.5.1, HK.3, BA.2.86 and JN.1 in comparison to WT, in infection-naïve vaccinees who received 4 doses of BNT or 3 doses of BNT162b2 boosted by a dose of BNT162b2(WT + BA.4/5). The bivalent vaccine elicited significantly higher neutralizing antibody levels to new omicron subvariants compared to boosting with the monovalent BNT162b2 vaccine. We found that BA.2.86 and JN.1 differed in neutralizing antibody evasion in infection-naïve BBB + BIV vaccinated sera. The continued monitoring for new variants that evade neutralizing antibody responses remains a priority.

## Data Availability

Data is provided within the manuscript or supplementary information files.
